# Self-assembly of 3D Ternary Crystals Consisting of Biological and Synthetic Nanoparticles

**DOI:** 10.1021/acs.biomac.6c00188

**Published:** 2026-05-27

**Authors:** Yu Zhou, Mauri A. Kostiainen

**Affiliations:** † Biohybrid Materials, Department of Bioproducts and Biosystems, Aalto University, 00076 Aalto, Finland; ‡ School of Chemistry, Xi’an Key Laboratory of Sustainable Energy Material Chemistry, 12480 Xi’an Jiao Tong University, 710049 Xi’an, People’s Republic of China

## Abstract

Inorganic nanoparticles can be assembled into ordered superlattices, where their collective properties lead to new optical, magnetic, or catalytic behavior. Protein cages, known for their highly symmetric and uniform structures as well as propensity for crystallization, can bind or encapsulate nanoparticles and serve as self-assembly templates, facilitating the formation of ordered superlattices. While binary systems based on protein cages have been successfully demonstrated, examples of ternary crystals incorporating protein cages remain largely unexplored. Here, we study the electrostatic self-assembly of three-component nanoparticle arrays composed of two protein cages: cowpea chlorotic mottle virus and ferritin as well as synthetic positively charged gold nanoparticles. We demonstrate that they can self-sort into binary crystals or form ternary complexes by tuning the particle interactions through the solution ionic strength. The cationic gold particles play a crucial role in modulating the crystallization process through electrostatic interactions, significantly impacting the formation of the nanoparticle array.

## Graphical abstract

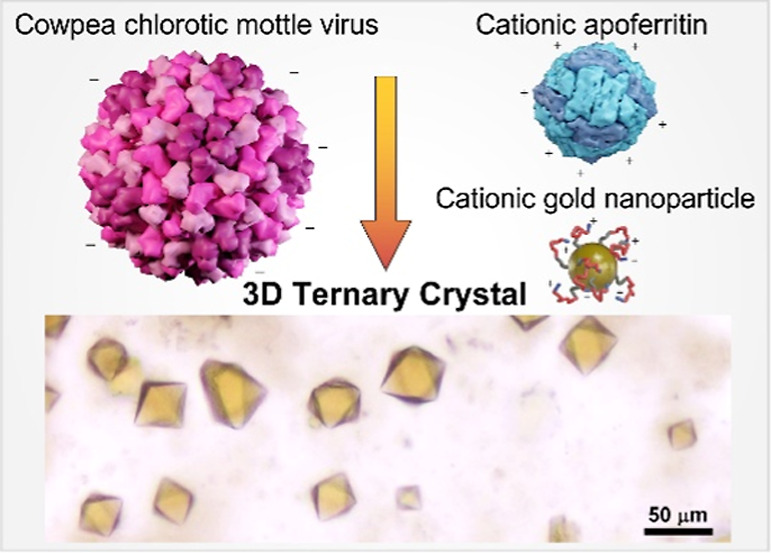

## Introduction

The assembly of nanocrystals into highly ordered superlattices is critical to the development of new functional materials for applications^1,2^ in electronics,^3–7^ optics,^8–11^ and catalysis.^12–16^ The unique collective behavior arising from the precise arrangement of constituent units can yield emergent properties not present in individual components. While substantial progress has been made in the synthesis of binary nanocrystal superlattices,^9,17–24^ the exploration of ternary systems remains comparatively limited.^25–29^ Ternary assemblies enable nanostructures with interactions between three different components, which can lead to high-performance materials such as ultrabright light sources^29^ and heteronanoplatelet catalysts.^30^ Therefore, understanding the principles governing the formation of ternary superlattices is essential to advance materials integrating multiple functionalities.

Protein cages,^31–37^ such as virus capsids and ferritins, serve as versatile templates for assembling nanoparticles,^38–45^ providing high uniformity and stability in superlattice formation.^46–49^ These biocompatible nanostructures enable precise organization of nanoparticles within ordered arrays, making them ideal candidates for fabricating high-quality crystals. While binary assemblies based on protein cages have shown potential for creating large superlattices, reports of such systems remain relatively rare.^43,50,51^ Moreover, studies on ternary assemblies involving protein cages are lacking. This presents a significant opportunity to investigate how interactions between three distinct nanoscale components mediated by protein cages can govern the assembly of higher-order nanoparticle structures. These effects are crucial for both understanding fundamental assembly mechanisms and developing applied nanomaterials.

In this work, we present a ternary array composed of cowpea chlorotic mottle virus (CCMV), ferritin and positively charged gold nanoparticles (pAuNPs), arranged into an ordered structure that retains the topological features of the binary protein cage system. The inclusion of pAuNPs plays a crucial role in modulating the crystallization process, as their electrostatic interactions with the protein cages influence the stability and spatial organization of the assembly. Notably, time-resolved synchrotron X-ray scattering results demonstrate that pAuNPs can influence the superlattice formation pathway, guiding the development of the ternary structure. Through careful control of component ratios and ionic strength, we systematically explore how these variables affect the structural characteristics of the ternary crystal, offering new insights into the assembly of ternary nanostructures.

## Methods

### Dynamic Light Scattering

A dynamic light scattering (DLS) device (Zetasizer Nano ZS Series, Malvern Instruments) was utilized to measure the hydrodynamic diameter (*D*_h_) of the assemblies. The device was equipped with a 4 mW He–Ne laser operating at a wavelength of 633 nm and an avalanche photodiode detector positioned at an angle of 173°. All experiments were conducted at 25 °C using PMMA cuvettes. The Zetasizer software (Malvern Instruments) was employed to analyze the scattering intensity (count rate) and determine particle size distributions.

In the experiments, CCMV (0.1 mg mL^–1^) was added to either an acetate buffer (20 mM sodium acetate, pH 4.9) or a Tris buffer (20 mM Tris, pH 7.4) containing cationic nanoparticles, either pAuNP (0.033 mg mL^–1^) and pFt (0.067 mg mL^–1^) or pAuNP (0.067 mg mL^–1^) and pFt (0.033 mg mL^–1^). For the dynamic measurements, a Tris buffer (20 mM Tris, pH 7.4) with 200 mM NaCl was used, with a monitoring duration of 15 min.

### In-house Small-Angle X-ray Scattering Experiments

The Xenocs Xeuss 3.0 C device, equipped with a GeniX 3D Cu microfocus source (with a wavelength of λ = 1.542 Å), and an EIGER2 R 1M hybrid pixel detector, was used to measure the SAXS samples. The measurements were conducted at a sample-to-detector distance of 1.1 m. To obtain one-dimensional SAXS data, the 2D scattering data was azimuthally averaged. The magnitude of the scattering vector *q* can be calculated using the equation *q* = 4π sin θ/λ, where 2θ represents the scattering angle.

For sample preparation, 6 μL of required NaCl solution (in 20 mM Tris–HCl, pH 7.4) was added to 1 μL of a cationic particle solution (either final concentration of pAuNP at 0.533 mg mL^–1^ and pFt at 1.067 mg mL^–1^ or vice versa) to adjust the ionic strength. Following this, 1 μL of CCMV solution (final concentration 1.6 mg mL^–1^) was added while stirring.

### Electron Microscopy Imaging

Conventional transmission electron microscopy (TEM) imaging of individual protein cages was performed using a Tecnai 12 Bio-Twin transmission electron microscope (FEI) at an acceleration voltage of 120 kV. The sample was applied to Formvar/Carbon-supported copper grids, and excess material was removed using filter paper. The grids were then washed with Milli-Q water, followed by one wash and a 30 s incubation in 2% uranyl acetate drops. Uranyl acetate incubations were omitted for unstained samples.

Cryogenic transmission electron microscopy (Cryo-TEM) images were obtained using a JEM 3200FSC field emission microscope (JEOL) operating at 300 kV in bright field mode and equipped with an Omega-type zero-loss energy filter. Gatan Digital Micrograph software was used to capture the images while maintaining the specimen temperature at −187 °C.

For Cryo-TEM sample preparation, a 3 μL aliquot of the aqueous dispersion (the same sample preparation as for in-house SAXS experiments) was deposited onto a 200-mesh Lacey carbon film supported on copper TEM grids (Agar Scientific). The grids were rapidly frozen by plunging them into liquid ethane using a Leica grid plunger. The blotting process lasted for 3 s under 100% humidity. The grids containing the vitrified sample solution were then maintained at liquid nitrogen temperature and transferred to the microscope under cryogenic conditions. Prior to use, all TEM grids underwent plasma cleaning using a NanoClean 1070 (Fischione Instruments). ImageJ software was employed for further processing of the acquired images.

### Optical Microscopy

Samples for optical microscopy were prepared using the hanging drop technique. A coverslip (18 × 18 mm, VWR) served as a platform to support a droplet suspended above the reservoir media, which consisted of 300 μL of 20 mM Tris buffer at pH 7.5. The droplet contained the same components used for SAXS measurements. To prevent solvent evaporation, the coverslip with the sample was secured to the reservoir cap using high vacuum glue, with the droplet facing the sealed side. The samples were then incubated at room temperature before imaging.

Imaging was conducted using a Zeiss Axio Vert A1 inverted microscope. The samples were directly imaged on the glass slides used during the crystallization process, without any additional preparation.

### Synchrotron SAXS Measurements

In situ SAXS characterization of the ternary superlattice formulation involving CCMV, pAuNP, and pFt was performed at the CoSAXS beamline at MAX IV. The experiments utilized an X-ray wavelength of 1 Å with a focused beam size of 50 × 60 μm^2^ directed at the sample, with data collected using an Eiger2 4M SAXS detector positioned 1.5 m from the sample.

A custom sample holder was employed to mount the microfluidic chip, maintaining the temperature at 24.5 ± 0.2 °C throughout the measurements. SAXS data were acquired at 24 distinct locations along the microfluidic channel: three along the herringbone geometry and 21 at the outlet. At each site, 40 frames were recorded with a 50 ms exposure time to monitor structural changes during the formation of the ternary superlattices.

The mixing process involved combining CCMV (0.1 mg mL^–1^), pAuNP (0.033 mg mL^–1^), and pFt (0.067 mg mL^–1^) in a 20 mM Tris buffer at pH 7.4 containing 200 mM NaCl using a Fluidic 187 Herringbone Mixer (COP) from Microfluidic ChipShop. A NEMESYS low-pressure syringe pump facilitated the process, set to a total flow rate of 10 μL/min for precise control over the formulation conditions. The microfluidic channel dimensions included a depth of 0.2 mm, an inlet width of 0.3 mm, and a mixing/outlet width of 0.6 mm.

## Results and Discussion

To explore the self-assembly of protein cage based ternary arrays, four distinct types of nanoparticles were initially selected, from which combinations of three were employed, each chosen for their complementary structural and functional properties. The largest protein nanoparticle used here, CCMV, is a spherical plant virus with an outer diameter of 28 nm (Figures 1a,b, S1a). The capsid is composed of 180 identical coat protein subunits arranged in Caspar–Klug *T* = 3 quasi-icosahedral symmetry, encapsulating multipartite positive-sense single-stranded RNA (+ssRNA) genome, totaling approximately 8000 nucleotides.^52^ The second nanoparticle, horse spleen apoferritin (aFt), is a naturally occurring protein cage involved in iron storage and mineralization (Figures 1c, S1b).^53^ Ferritin assembles into a hollow, spherical shell with octahedral symmetry, featuring an outer diameter of 12 nm and an inner cavity diameter of 8 nm.^54^ Both CCMV and aFt exhibit a net negative surface charge at neutral pH (isoelectric points of 3.8 ^55^ and 4.5,^56^ respectively). As the third particle, a positively charged ferritin variant (pFt) was engineered by introducing nine lysine/arginine residues per subunit (Figures 1d and S1c),^57^ enabling electrostatic interactions with negatively charged protein cages at neutral pH. The last component, positively charged gold nanoparticles (pAuNPs), were stabilized by an amphiphilic ligand containing a cationic quaternary amine end-group. The pAuNPs possess a metallic core with an average diameter of 2.6 nm, and the ligand-stabilized particles have an overall diameter of approximately 8.5 nm (Figures 1e and S1d).

**Figure 1:**
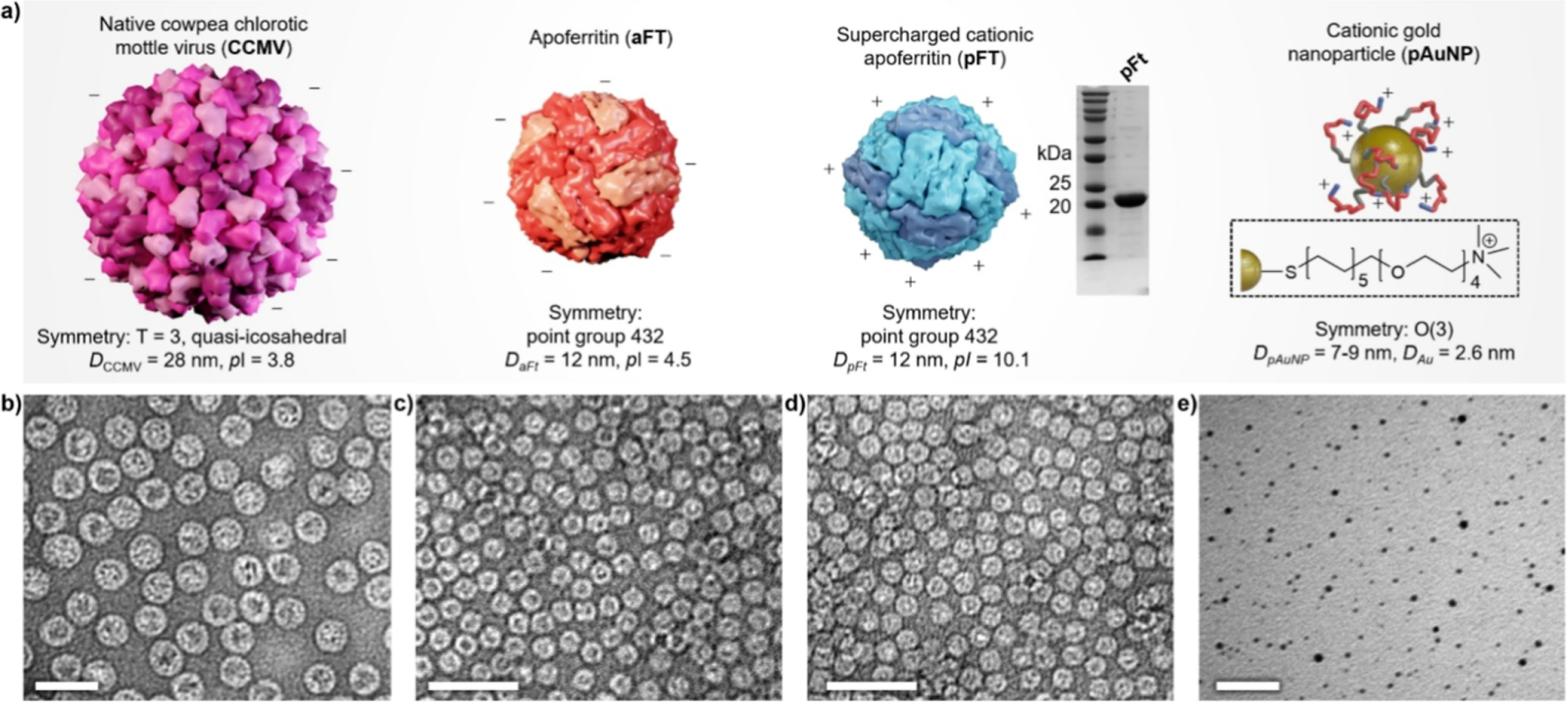
Materials used for the assembly of protein cage-nanoparticle arrays. (a) Constituent building blocks for array construction: native cowpea chlorotic mottle virus (CCMV), apoferritin (aFt), supercharged cationic apoferritin (pFt) as well as its SDS-PAGE analysis, and cationic gold nanoparticle (pAuNP). TEM images of: (b) CCMV, (c) aFt, (d) pFt and (e) pAuNP. All scale bars: 50 nm.

Different combinations of positively and negatively charged nanoparticles were first selected from the set of four particles as controls. The four pairwise combinations have been characterized in previous studies (Figure 2a).^43,45,58^ We then proceeded to examine ternary combinations, which were first studied with small-angle X-ray scattering (SAXS) at pH 7.4 with 190 mM NaCl. Analysis of the scattering pattern indicates that the pFt–aFt–CCMV protein-only complexes exhibit a face-centered cubic (FCC) Bravais lattice (space group Fm3̅m, number 225) with a lattice parameter of *a* = 48.3 nm (Figure 2b). The absence of the (220) reflection (*q* ≈ 0.035 Å^–1^) does not contradict the FCC assignment, as it coincides with the CCMV form factor minima. The scattering pattern is clearly similar to the previously investigated binary combinations of pFt and gold nanoparticle–loaded CCMV^43^ also forming a FCC lattice. In the ternary system at pH 7.4, aFt is negatively charged and able to interact with positively charged pFt. This transient effect may modify the electrostatic attraction between pFt and negatively charged CCMV affecting the assembly pathway. In the protein-only ternary system, the particle array components are difficult to determine and thus the role of aFT cannot be directly resolved.

**Figure 2:**
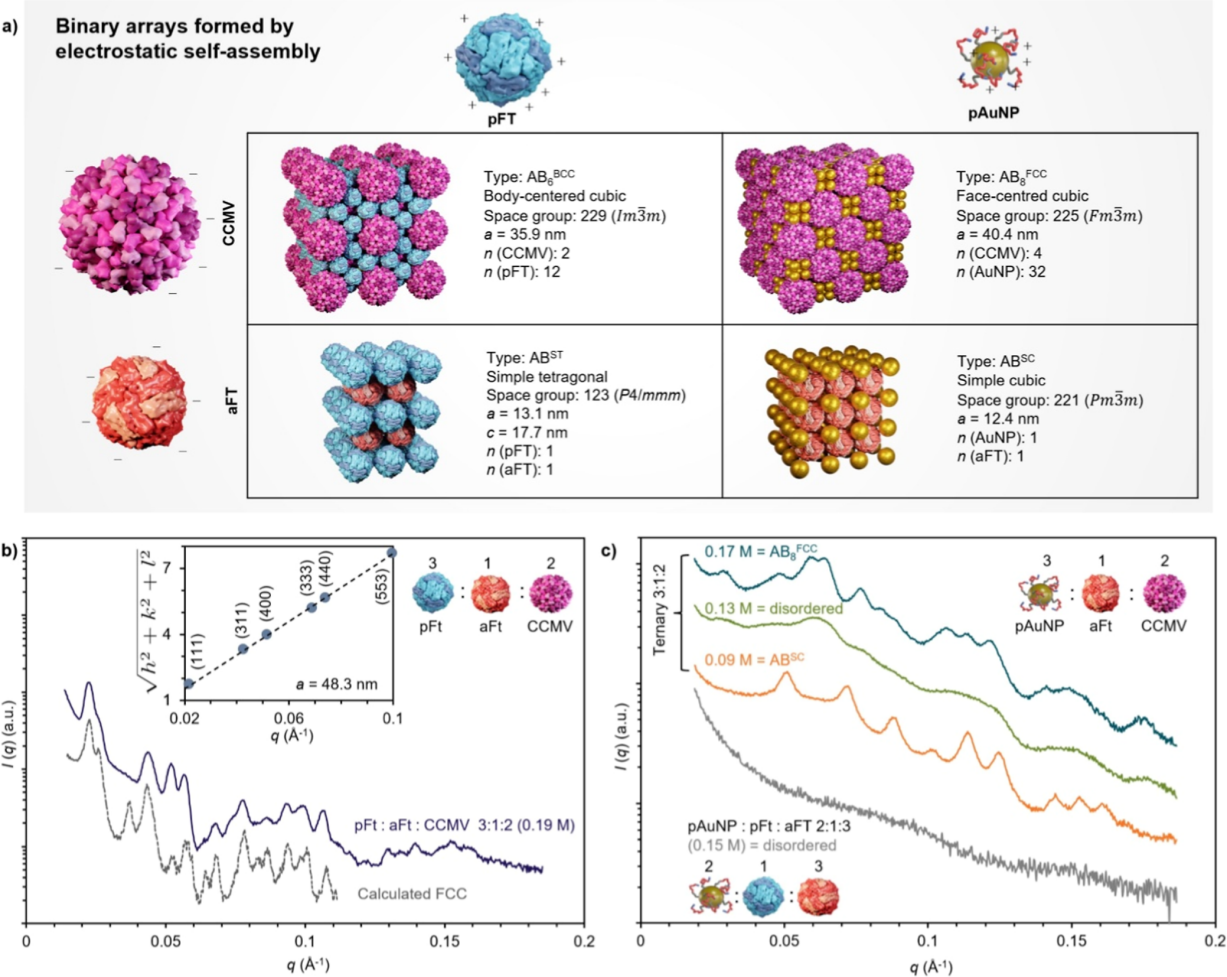
Structural details of the nanoparticle assemblies. (a) Binary arrays formed by electrostatic self-assembly. (b) SAXS of pFt–aFt–CCMV complexes at 190 mM NaCl, compared to a simulated FCC model (vertical offset for clarity). Inset: square root of the sum of the square of the Miller indexes of the assigned reflections for the FCC structure versus the measured *q*-vector positions, dashed line presents a linear fit, which yields a lattice parameter *a*_SAXS_ = 48.3 nm (for cubic structures *a* = 2π √(*h* ^2^ + *k* ^2^ + *l* ^2^)/*q*_(*hkl*)_). (c) Structural characterization of pAuNP–pFt–aFt and pAuNP–aFt–CCMV complexes at various NaCl concentrations.

To have more distinguishable components, we proceeded to the subsequent investigation of pAuNP–aFt–CCMV samples, prepared at a 3:1:2 weight ratio (w/w/w). At a relatively low NaCl concentration of 0.09 M, the SAXS pattern revealed intriguing self-sorting behavior, corresponding exclusively to interpenetrating simple cubic pAuNP-aFt assemblies with *a* = 12.9 nm. In contrast, at a higher NaCl concentration of 0.17 M, the SAXS pattern shows only the assembly of binary AB_8_^FCC^ pAuNP-CCMV arrays with *a* = 41.1 nm (Figure 2c). Both of these lattices have been previously observed in pure binary systems.^45^ Here, the self-sorting phenomenon can be attributed to the different electrostatic interactions between pAuNPs, aFt, and CCMV under varying salt concentrations. At 0.09 M NaCl, the significantly higher molar ratio of aFt to CCMV (approximately 4.6:1) allows pAuNPs to preferentially interact with aFt particles, facilitating rapid assembly. In contrast, at 0.17 M NaCl, the electrostatic interactions between pAuNPs and aFt are weakened and partially screened by the salt, enabling CCMV to assemble with pAuNPs due to their relatively stronger electrostatic interactions (*p*I_CCMV_ < *p*I_aFT_).

Based on this observation, we hypothesize that if a well-ordered ternary crystal can be assembled from these particles, the two larger protein nanoparticles should serve as the structural scaffold for the ternary superlattice, while the smaller synthetic pAuNPs would act as intercalators, occupying the interstitial sites between the protein cages. However, the pAuNP–pFt–aFt combination did not form an ordered structure (Figure 2c), further highlighting the crucial role of CCMV in stabilizing ternary assemblies. Therefore, in the following section we systematically investigate the ternary combination of pAuNP–pFt–CCMV, to evaluate its potential for forming a well-defined superlattice.

Precise modulation of electrostatic interactions between nanoparticles by controlling ionic strength has proven effective in binary arrays formed by CCMV and pFt. To investigate the formation of ternary complexes under different pH and salt conditions, dynamic light scattering (DLS) was first employed to track changes in scattering intensity (derived count rate) and hydrodynamic diameter (*D*_h_). In these experiments, CCMV (0.1 mg mL^–1^) was added to either an acetate buffer (20 mM sodium acetate, pH 4.9) or a Tris buffer (20 mM Tris, pH 7.4) containing both cationic nanoparticles: pAuNP (0.033 mg mL^–1^) and pFt (0.067 mg mL^–1^), corresponding to an estimated molar ratio of pAuNP/pFt/CCMV of approximately 19:6:1. Upon CCMV addition, a rapid increase in count rate was observed, indicating the formation of large complexes. The role of electrostatic interactions was confirmed by adding NaCl, which decreases the Debye screening length thereby reducing the electrostatic attraction between particles and ultimately the complex size. At pH 4.9, concentrations above 200 mM NaCl reduced the count rate to the original state, suggesting complete disassembly. By contrast, at pH 7.4, the aggregates remained relatively stable even at NaCl concentrations exceeding 500 mM, consistent with observations in the binary system lacking pAuNPs (Figure 3a). This behavior likely arises from overcrowding of pFt particles around CCMV at neutral pH, forming irreversible complexes.^59^ Further evidence of reversible assembly at pH 4.9 was obtained from *D*_h_ measurements. After CCMV addition, *D*_h_ increased to 1471 nm, while the peak at 13.3 nm representing the size of the free cationic particles diminished. Following the introduction of 240 mM NaCl, the *D*_h_ reverted to 30 nm, indicating successful disassembly of the ternary complex and the return of all particles to their original free state (Figure 3b). As with the pFt-CCMV binary system,^43^ reversible assembly of the ternary complex at neutral pH could be regulated by adjusting the order of component addition. This strategy was subsequently adopted for all further measurements to ensure consistency and accuracy. As shown in Figure 3c, a solution of cationic particles was first prepared in a neutral pH buffer with a predetermined NaCl concentration. Introducing NaCl prior to mixing the oppositely charged particles slowed the assembly process, preventing the formation of kinetically trapped aggregates. After the system stabilized, CCMV was added to a final concentration of 0.1 mg mL^–1^. As expected, large complexes (*D*_h_ > 1000 nm) were observed in solutions containing 150 mM NaCl, whereas only free particles were detected at NaCl concentrations above 400 mM.

**Figure 3:**
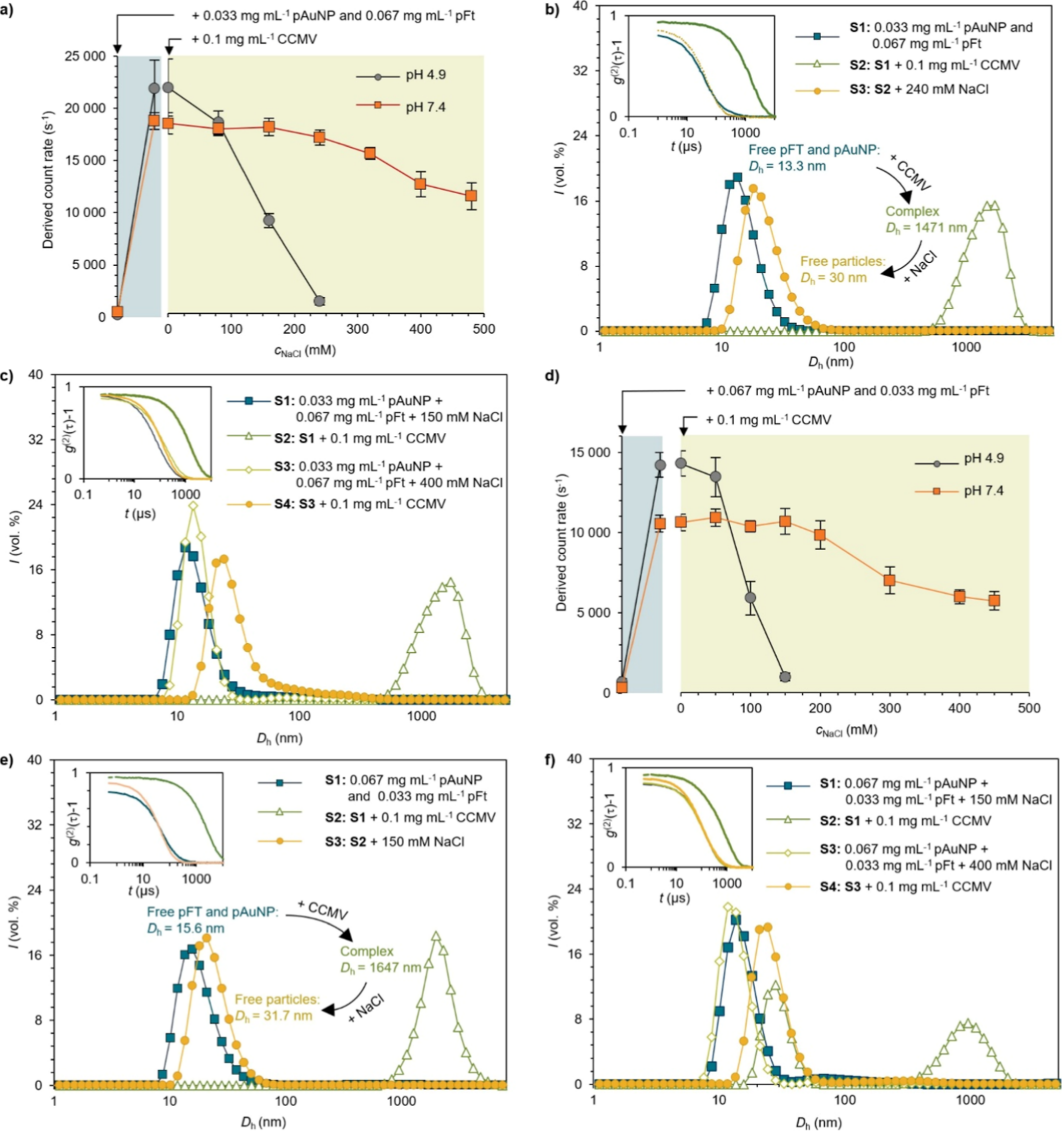
Self-assembly of ternary complexes at pH 4.9 and pH 7.4. (a) Left: light scattering intensity of a cationic nanoparticle solution (pAuNPs at 0.033 mg mL^–1^ and pFt at 0.067 mg mL^–1^) upon the addition of CCMV solution (0.1 mg mL^–1^), indicating complex formation. Right: complexes were disassembled by increasing the ionic strength of the medium through the addition of NaCl at pH 4.9. However, at pH 7.4 the complexes remained relatively stable. (b) DLS data showing the volume-averaged size distribution of complex formation and disassembly at pH 4.9 from panel (a). Inset shows the corresponding second order normalized autocorrelation functions. (c) DLS characterization of the ternary assembly at pH 7.4 with a different sequence of adding materials: the cationic nanoparticles were first dispersed in the pH 7.4 buffer with the desired concentration of NaCl before the introduction of CCMV into the system. In this manner, 400 mM NaCl is sufficient to disassemble the complexes through increased charge screening. (d–f) The same DLS measurement for a cationic nanoparticle solution (pAuNPs at 0.067 mg mL^–1^ and pFt at 0.033 mg mL^–1^) upon the addition of CCMV solution (0.1 mg mL^–1^). Measurements were performed at pH 4.9 for (e), and at pH 7.4 for (f).

To confirm that the assembly process is not restricted to a narrow range of particle ratios, a different concentration of AuNPs (0.067 mg mL^–1^) and pFt (0.033 mg mL^–1^) was mixed with CCMV (0.1 mg mL^–1^), corresponding to an estimated molar ratio of pAuNP: pFt/CCMV of approximately 38:3:1. The DLS data showed a similar trend as with the initial particle concentrations, although the resulting aggregates were more sensitive to NaCl addition. At pH 4.9, complete disassembly occurred at a lower NaCl concentration of 150 mM (Figure 3d,e). This is further supported by the *D*_h_ data in Figure 3f, which shows two distinct peaks at 150 mM NaCl: one corresponding to large aggregates (*D*_h_ > 1000 nm) and the other to free particles. The presence of both peaks suggests partial disassembly of the aggregates at this NaCl concentration.

Small-angle X-ray scattering (SAXS) measurements were performed on samples with varying ratios of cationic nanoparticles to CCMV and different NaCl concentrations. Photographs of the SAXS capillary samples showed that mixtures containing pAuNPs formed a distinct red–colored complex, indicating the successful incorporation of pAuNPs into the assemblies (Figure S2). Initially, a ternary system of pAuNPs, pFt and CCMV (1.6 mg mL^–1^) in a 1:2:3 weight ratio was prepared with different NaCl concentrations for SAXS analysis (Figure 4a). However, the scattering patterns in the ternary samples prepared by direct mixing were not sufficiently pronounced to allow structure determination (data not shown).

**Figure 4:**
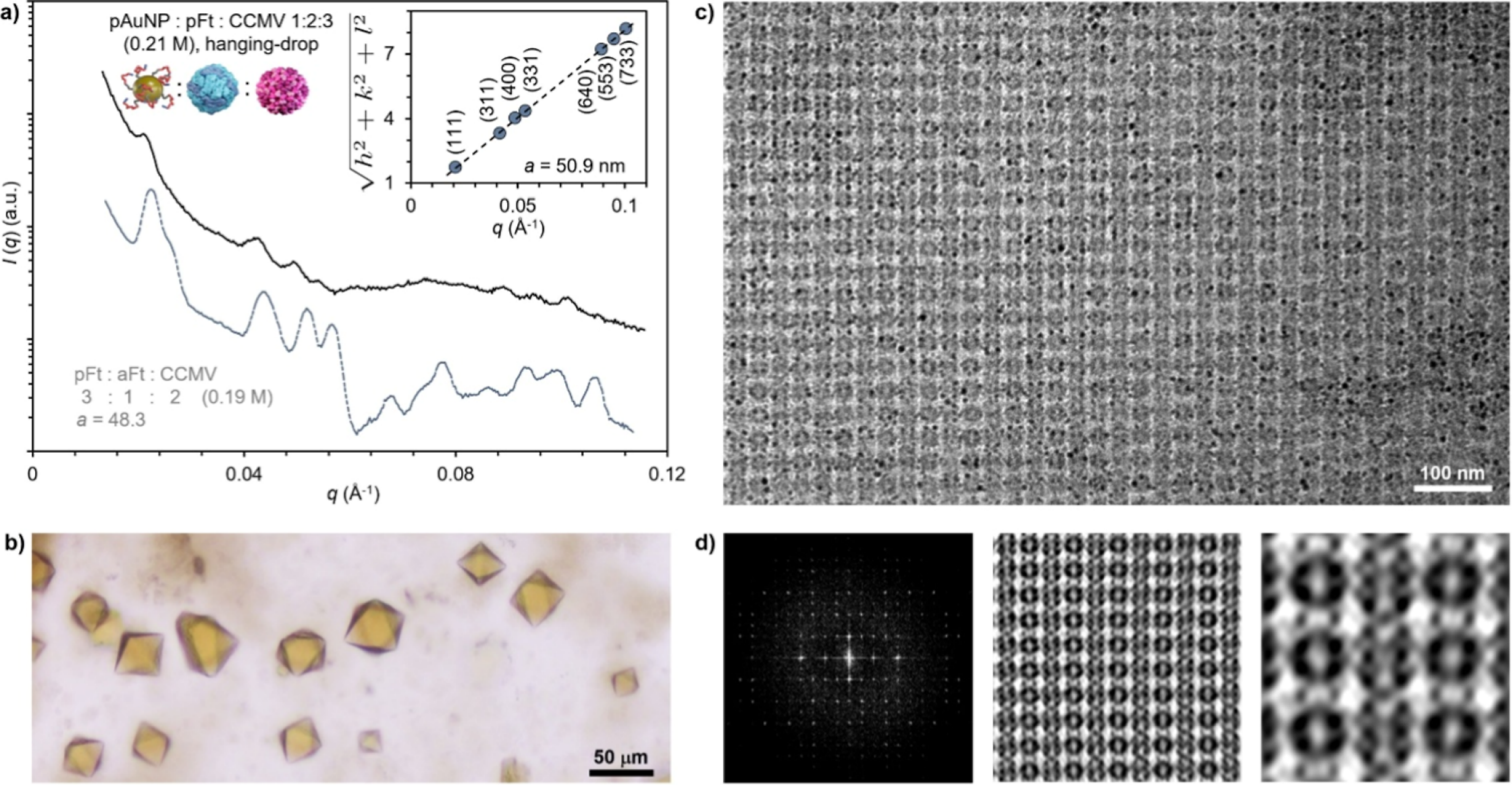
Structural characterization of the ternary complexes. (a) Integrated one-dimensional SAXS data of the 1:2:3 ternary complex prepared using the hanging-drop setup at 210 mM NaCl, compared to the scattering measured from the pFt/aFt:CCMV 3:1:2 sample in Figure 2b. (b) Optical microscopy image of 1:2:3 ternary crystals obtained with a hanging drop setup, showing crystals with dimensions exceeding 20 μm. (c) Cryo-TEM image of 1:2:3 ternary complex at 210 mM NaCl showing an ordered protein structure, but relatively heterogeneous distribution of AuNPs. (d) Fast Fourier transform (FFT) (left), 300 nm × 300 nm inverse FFT calculated using selected Fourier components (middle) and a 100 nm × 100 nm high magnification version (right), all calculated from the image in panel (c).

We therefore prepared 1:2:3 ternary crystals using a hanging drop setup. By comparing the SAXS data of the 1:2:3 ternary complex at 210 mM NaCl with the data of the pFt/aFt/CCMV 3:1:2 ternary complex at 190 mM NaCl, we can observe several similar characteristic peaks (Figure 4a). The four most prominent peaks occur at *q* = 0.02098 Å^–1^, 0.04181 Å^–1^, 0.04904 Å^–1^, and 0.05376 Å^–1^. From these findings, we propose that the 1:2:3 ternary structure likely adopts a FCC structure with a lattice constant of *a* = 50.9 nm. Optical microscopy images (Figures 4b and S3) confirmed the formation of large well-defined crystals with apparent octahedral shape, which is a typical habit for protein cage crystals.^60,61^ However, the SAXS patterns are weaker than expected for crystals of this size, likely due to uneven occupancy of pAuNP lattice sites within the ternary crystal.

To further investigate the distribution of pAuNPs within the ternary complexes and their contribution to the overall structure, cryogenic transmission electron microscopy (cryo-TEM) was employed. Figures 4c and S4 shows the 1:2:3 complex at 210 mM NaCl revealing large crystalline structures with sizes of several micrometers. Notably, although the protein particles appear to be highly ordered, pAuNPs were observed to be relatively sparsely distributed throughout the crystalline structure. Inverse Fourier transform calculation using selected Fourier components show that the pAuNPs seem to occupy mostly defined lattice sites (Figure 4d). This apparent local randomness can be explained by partial occupancy: pAuNPs may occupy several symmetry-equivalent interstitial sites, but not all sites are filled in every unit cell, leading to an interstitial-type solid that appears locally disordered yet yields a coherent Fourier signal. However, the precise distribution of pAuNPs within the FCC lattice remains to be resolved.

To explore the potential effect of pAuNPs on the crystallization kinetics, DLS measurements were initially conducted to monitor the rate of complex formation in the presence of 200 mM NaCl and neutral pH at different weight ratios, by tracking the increase in derived count rate over time. In both the 1:2:3 and 2:1:3 ratios (0.1 mg mL^–1^ CCMV), complex formation occurred rapidly, with a noticeable increase in count rate within just a few tens of seconds after mixing, reaching a plateau after approximately 3 min. On this time scale, the presence of pAuNPs had no discernible impact on the rate of complex formation (Figure 5a,b).

**Figure 5:**
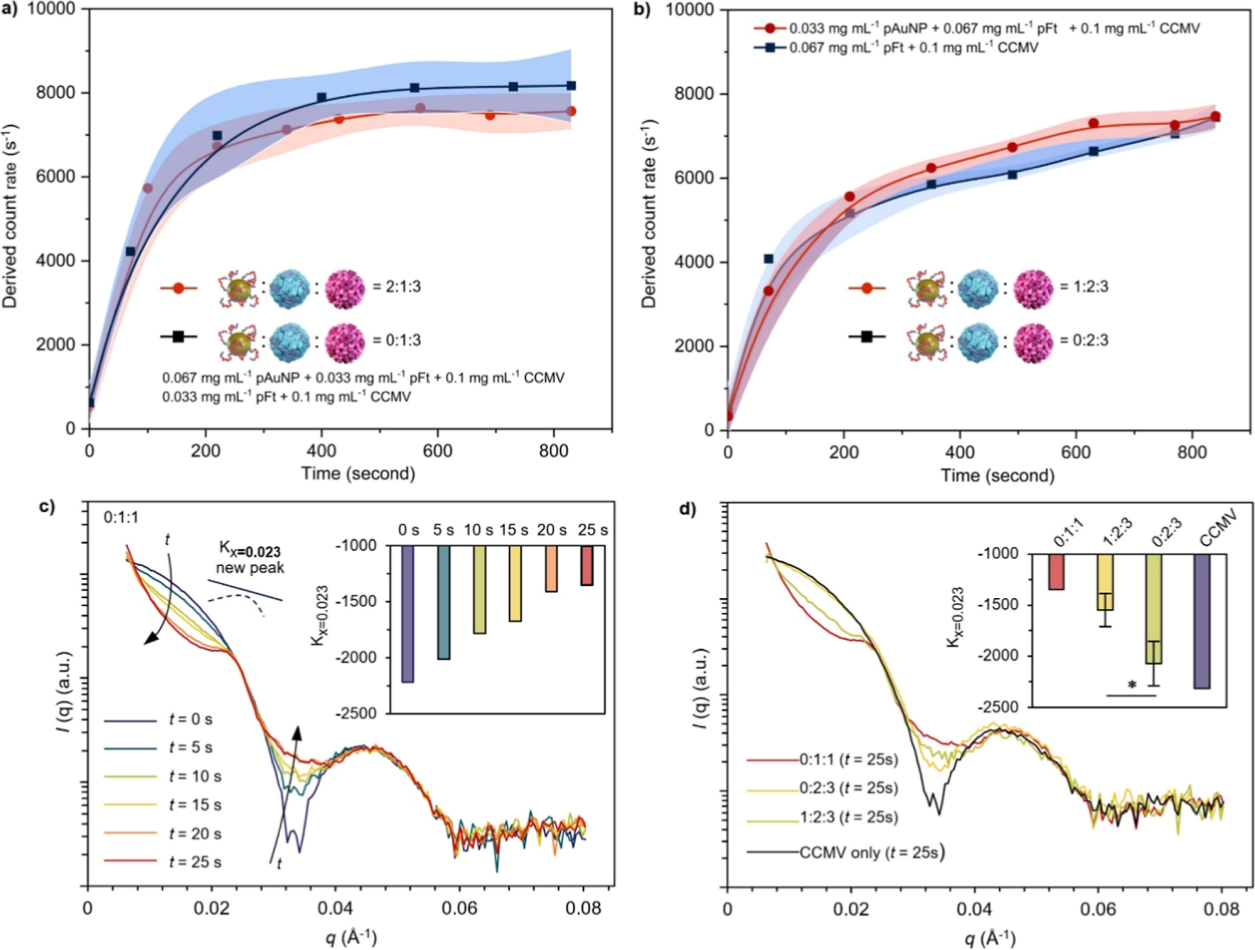
Characterization of assembly kinetics for ternary and binary complexes. (a,b) Assembly kinetics monitored by DLS measurements for the 1:2:3 and 2:1:3 ternary complex formation at 200 mM NaCl. (c) Assembly kinetics of 0:1:1 binary complex at 200 mM NaCl monitored by synchrotron SAXS over 25 s. (d) Comparison of the assembly kinetics of 0:1:1 and 0:2:3 binary complexes with 1:2:3 ternary complexes at 200 mM NaCl, monitored by synchrotron SAXS at the 25 s mark. The inset bar graphs in (c,d) show the slope of the tangent at *q* = 0.023 Å^–1^, which is used to monitor the assembly kinetics. Error bars represent the standard deviations from three independent measurements.

By contrast, synchrotron SAXS in a microfluidic setup enabled probing assembly dynamics on the timescale of seconds, providing deeper insight into the influence of pAuNPs. As a control, the formation kinetics of 0:1:1 binary complex (1.6 mg mL^–1^ CCMV) without pAuNPs was tracked over 25 s. A clear peak around *q* = 0.024 Å^–1^ emerged, corresponding to the initial formation of the crystal nucleus (Figure 5c). Similarly, dynamic synchrotron SAXS measurements were performed for the 0:2:3 and 1:2:3 samples (1.6 mg mL^–1^ CCMV). The SAXS curve for the 0:2:3 sample, which lacked pAuNP, closely resembled the form factor of free CCMV particles, indicating slower assembly, potentially due to the lower concentration of pFt, which in turn slowed the formation of the binary structure. However, in the 1:2:3 sample containing pAuNP, the assembly process accelerated, and the resulting SAXS curve showed a pronounced peak at *q* ≈ 0.023 Å^–1^, similar to that observed in the 0:1:1 sample (Figure 5d). We used the slope of the tangent at 0.023 Å^–1^ to monitor the assembly dynamics. This analysis revealed that the 1:2:3 weight ratio sample exhibited a significantly faster assembly into higher-order structures. These results suggest that the introduction of pAuNPs enables rapid assembly of well-defined ternary FCC arrays under conditions where the pFt concentration alone is insufficient for ordering (0:2:3). Simply increasing the pFt concentration (0:1:1) leads to faster kinetics but yields a binary BCC structure, not the ternary FCC lattice. Thus, pAuNPs provide a specific pathway to ternary coassembly by serving as interstitial mediators.

Building on our previous work, we demonstrate that CCMV and pAuNPs coassemble into an AB_8_-type FCC binary superlattice,^45^ whereas CCMV together with pFt cages forms an AB_6_-type BCC lattice.^43^ Remarkably, when all three components are combined in solution, they synergistically direct the formation of a ternary superlattice, with each building block affecting the interparticle spacing, packing symmetry, and ultimately the crystal architecture. We observe that here pAuNPs play a critical role in facilitating the formation of ternary structures by optimizing spatial organization, selectively occupying voids within the crystal lattice and enhancing the packing of larger CCMV and pFt particles. Furthermore, the electrostatic interactions introduced by pAuNPs may stabilize the assembly and promote ordering by preventing kinetically trapped structures, enabling the formation of well-defined ternary lattices even at relatively low pFt concentrations.

## Conclusions

This study reports the formation of a ternary lattice composed of CCMV, pFt, and pAuNPs. Building on prior work on binary protein-cage lattices, we tuned the ionic strength to control crystallization, yielding structures with long-range translational order. SAXS and cryo-TEM show that pAuNPs facilitate the formation of the ternary lattice under conditions where pFt alone is insufficient for rapid ordering. Our findings indicate that introducing pAuNPs plays a critical role in facilitating ternary structure formation. This research emphasizes the principle that strategically incorporating additional nanoparticle components can modulate assembly dynamics, paving the way for future material design. Ultimately, this work opens new avenues for engineering functional metamaterials by manipulating superlattice structures.

## Supplementary Material



## Notes

Description of materials, pFt protein expression and purification, estimation of particle molar ratios, protein sequences and additional TEM images of the used particles, photographs of SAXS samples, optical microscopy images of crystals and cryo-TEM images of the pAuNP/pFt:CCMV 1:2:3 complexes (PDF)
