# Supplementary material for: Self-assembly of 3D Ternary Crystals Consisting of Biological and Synthetic Nanoparticles
Source: Biomacromolecules. 2026 May 27;27(6):3787–95. doi: 10.1021/acs.biomac.6c00188 (PMC13250914; doi:10.1021/acs.biomac.6c00188)
Supplement: Supplementary file 1 [file bm6c00188_si_001.pdf]

Self-Assembly of 3D Ternary Crystals Consisting of Biological and Synthetic Nanoparticles

*Yu Zhou[1,2], and Mauri A. Kostiainen[1,]**

[1] Biohybrid Materials, Department of Bioproducts and Biosystems, Aalto University, 00076 Aalto, Finland.

[2] School of Chemistry, Xi'an Key Laboratory of Sustainable Energy Material Chemistry, Xi'an Jiao Tong University, 710049 Xi'an, People's Republic of China.

E-mail: mauri.kostiainen@aalto.fi

Materials. CCMV was produced and isolated from California black-eyed peas (*Vigna unguiculata*).[1] Horse spleen derived apoferritin (aFt) was purchased from Sigma-Aldrich and received in 0.135 M NaCl solution, further diluted in milliQ water. The synthesis of the cationic gold nanoparticles (pAuNP) was carried out as previously reported.[1] Glass microscope slides and cover slips were obtained from VWR. All other chemicals were sourced from Sigma Aldrich, unless otherwise specified. MilliQ water with a resistivity ≥ 18.2 MΩ·cm was used for all experiments.

pFt Protein Expression and Purification. The pFt backbone sequence was first optimized using OptimumGene™ and subsequently synthesized and cloned into the pET-25b(+) vector by GenScript. The *E. coli* T7 Express strain (New England Biolabs) was selected as the expression host. Protein expression and purification were conducted following a previously established protocol.[2] The purity of the protein was analyzed by SDS-PAGE, and its concentration was determined using a NanoDrop LITE spectrophotometer (Thermo Scientific).

Estimation of molar ratios. The number density of pAuNPs was estimated from absorbance at 450 nm to be approximately 7–8 × 10^{15} NPs/mg. The molecular weights of CCMV and pFt are 4.6 MDa and 480 kDa, respectively. Based on these values, the following pAuNP:pFt:CCMV ratios are calculated: for the mass ratio 1:2:3 (0.033 mg mL^{-1} pAuNP, 0.067 mg mL^{-1} pFt, 0.1 mg mL^{-1} CCMV), the molar ratio is approximately 19:6:1; for the mass ratio 2:1:3 (0.067 mg mL^{-1} pAuNP, 0.033 mg mL^{-1} pFt, 0.1 mg mL^{-1} CCMV), the molar ratio is approximately 38:3:1. The molar ratios should be treated as rough estimates due to the

uncertainty in determining the number density of small pAuNPs. However, the mass ratios can be accurately measured.

Protein Sequences. The pFt sequence used in this study was derived from FtnQC12, with nine mutated residues highlighted in red.

MTTASTSQVRQNYHQDSEKAINRQIRLELYASYVYLSMSYYFDRDDVALKNFAKYFL
HQSHEEREHAEKLMKLQNQRGGRIFLQDIQKPDKDDWESGLRAMEKALKLEKKVN
QSLLELHKLATKKNDPHLCDFIETHYLNEQVKAIKELGDHVTNLRKMGAPRSGLAEY
LFDKHTLGDSDNES

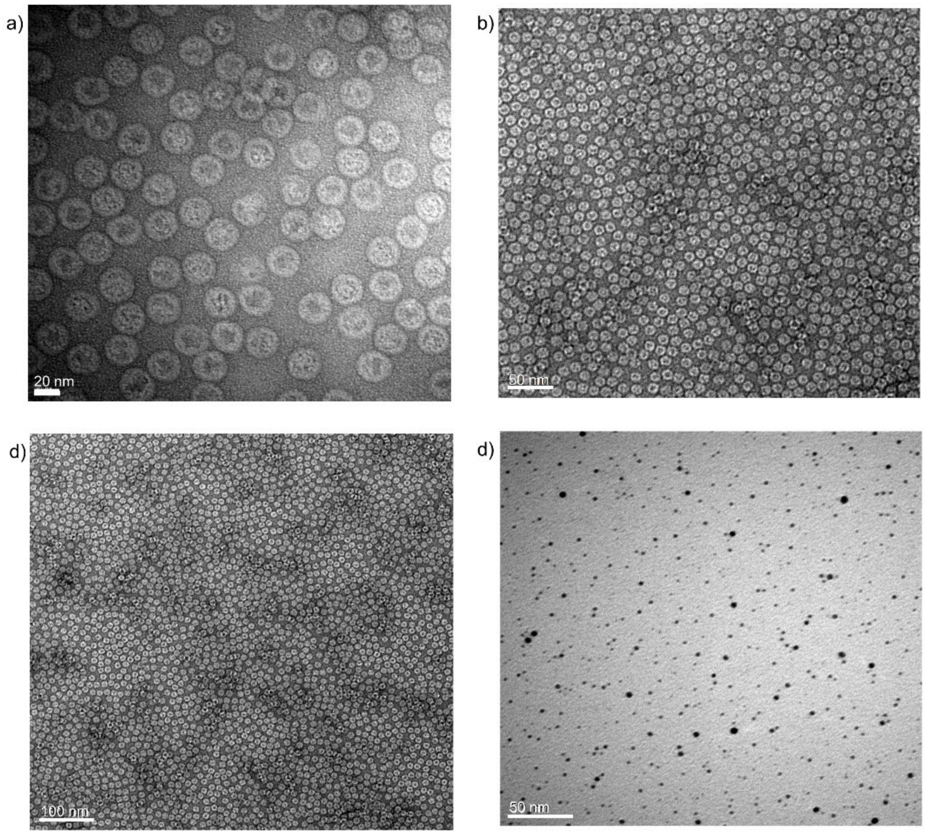

Figure S1. TEM analysis of (a) native CCMV in pH 4.9 buffer, scale bar is 20 nm. (b) aFt in pH 7.4 buffer, scale bar is 100 nm. (c) pFt in pH 7.4 buffer, scale bar is 50 nm. (d) pAuNPs in water, scale bar is 50 nm.

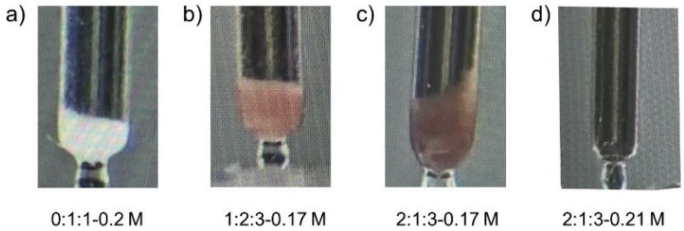

Figure S2. Photographs of pAuNP:pFt:CCMV SAXS samples in capillaries. (a) 0:1:1 complexes at 0.20 M NaCl. (b) 1:2:3 complexes at 0.17 M NaCl. (c) 2:1:3 complexes at 0.17 M NaCl. (d) 2:1:3 complexes at 0.21 M NaCl.

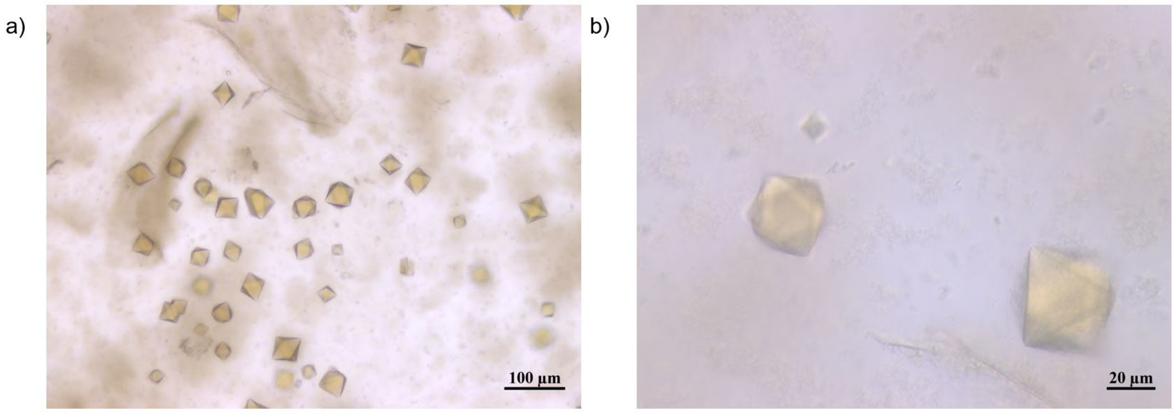

Figure S3. Additional optical microscopy image of pAuNP:pFt:CCMV 1:2:3 ternary crystals at 0.21 M NaCl obtained with a hanging drop setup.

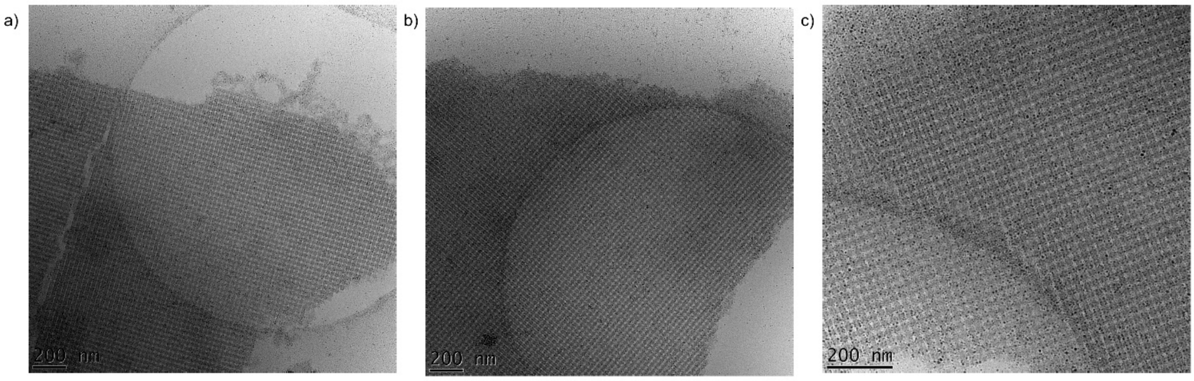

Figure S4. (a-c) Additional cryo-TEM images of the pAuNP:pFt:CCMV 1:2:3 complexes at 0.21 M NaCl, scale bars are 200 nm.

REFERENCES

[1] M.A. Kostiainen, P. Hiekkataipale, A. Laiho, V. Lemieux, J. Seitsonen, J. Ruokolainen, and P. Ceci, *Nat. Nanotechnol.* 2013, **8**, 52-56.

[2] M. Künzle, T. Eckert, and T. Beck, *J. Am. Chem. Soc.* 2016, **138**, 12731-12734.
